# Psychological inoculation can reduce susceptibility to misinformation in large rational agent networks

**DOI:** 10.1098/rsos.211953

**Published:** 2022-08-10

**Authors:** Toby D. Pilditch, Jon Roozenbeek, Jens Koed Madsen, Sander van der Linden

**Affiliations:** ^1^ School of Geography and the Environment, University of Oxford, South Parks Road, Oxford, OX1 3QY, UK; ^2^ Department of Psychology and Language Studies, University College London, Gower Street, London, WC1E 6BT, UK; ^3^ Cambridge Social Decision-Making Laboratory, Department of Psychology, School of Biology, University of Cambridge, Cambridge, CB2 3RQ, UK; ^4^ Department of Psychological and Behavioural Science, London School of Economics, Kings Way, London, WC2A 2AE, UK

**Keywords:** misinformation, inoculation theory, complex systems, belief updating

## Abstract

The unchecked spread of misinformation is recognized as an increasing threat to public, scientific and democratic health. Online networks are a contributing cause of this spread, with echo chambers and polarization indicative of the interplay between the search behaviours of users and reinforcement processes within the system they inhabit. Recent empirical work has focused on interventions aimed at inoculating people against misinformation, yielding success on the individual level. However, given the evolving, dynamic information context of online networks, important questions remain regarding how such inoculation interventions interact with network systems. Here we use an agent-based model of a social network populated with belief-updating users. We find that although equally rational agents may be assisted by inoculation interventions to reject misinformation, even among such agents, intervention efficacy is temporally sensitive. We find that as beliefs disseminate, users form self-reinforcing echo chambers, leading to belief consolidation—irrespective of their veracity. Interrupting this process requires ‘front-loading’ of inoculation interventions by targeting critical thresholds of network users before consolidation occurs. We further demonstrate the value of harnessing tipping point dynamics for herd immunity effects, and note that inoculation processes do not necessarily lead to increased rates of ‘false-positive’ rejections of truthful communications.

## 1. Introduction

The spread of misinformation—both online and offline—is a significant and growing societal challenge with negative impacts on science, democracy and public health [1]. For example, misinformation about climate change undermines people's confidence in the scientific consensus [2,3], disinformation spread on WhatsApp has led to mob lynchings [4], 5G conspiracy theories have motivated people to set phone masts ablaze [5], and susceptibility to misinformation has been linked to reduced willingness to get vaccinated against COVID-19 [6], potentially compromising future herd immunity [7]. In response, researchers have emphasized the role of behavioural science in mitigating the spread of misinformation [8]. Although a variety of approaches have been proposed to counter misinformation (see [9] for a recent review), a particularly prominent approach is to develop interventions (such as games, messages, infographics or short videos) that build psychological resistance against unwanted persuasion, an approach known as inoculation theory [10–12]. However, while the principle of cognitive inoculation has been shown to be effective at conferring psychological resistance against misinformation at the individual level, for example in the context of COVID-19 [13], political disinformation [14] and climate change [15], a crucial open question with significant theoretical and applied importance is to what extent ‘psychological herd immunity’ against misinformation is feasible [13,16]. To address this challenge, we integrate inoculation theory within an agent-based model (ABM) to explore the effects of applying psychological inoculation on the spread of misinformation, belief polarization and the formation of echo chambers [17–19]. ABMs allow for implementation of cognitive, social and structural elements related to the spread of misinformation and enable researchers to test the effectiveness of the inoculation model on a societal scale.

### 1.1. Inoculation theory

Misinformation is known to spread in social networks much like a viral pathogen. To capture this, models from epidemiology, such as the SIR (susceptible-infected-recovered) model, are increasingly used to study how misinformation may cascade [20–23]. One possible solution to the misinformation problem is to psychologically immunize people against misinformation [24]. Indeed, researchers have asked ‘how feasible it is to try to make people “immune” to misinformation’ [25, p. 1]. Inoculation theory is a model from social psychology inspired by the biomedical analogy [12,26]. Just as administering a weakened dose of a viral pathogen triggers the production of antibodies to help fight off future infection, inoculation theory posits that the same can be achieved with information. By pre-emptively exposing people to sufficiently weakened doses of a persuasive attack, immunity to persuasion can be conferred. Inoculation theory works through a process known as ‘prebunking’ (i.e. refuting false information in advance), which helps people fortify their cognitive defences. Meta-analyses have shown that inoculation theory is one of the most robust frameworks for countering the persuasive efficacy of misinformation [10].

Inoculation theory has only recently been applied to the context of misinformation, but applications have varied from immunizing people against misinformation about climate change [2,3,15], immigration [27] and extremism [28,29] to conspiracy theories [5] and misinformation about COVID-19 [13,30]. For reviews of this burgeoning literature, we refer to [16] and [11].

Typically, inoculation messages or campaigns forewarn people of an impending attack and pre-emptively refute specific myths. For example, to get ahead of voting misinformation, Twitter implemented a prebunking campaign based on inoculation theory in advance of the 2020 Presidential election where they forewarned all US users that they might come across false information about voting by mail on their platform. This inoculation contained a specific refutation; ‘Election experts confirm that voting by mail is safe and secure, even with an increase in mail-in ballots' [31].

However, it is difficult to ‘prebunk’ or ‘inoculate’ people against every single myth. For this reason, inoculation theory has recently seen major advances to improve scalability of the cognitive ‘vaccine’ by exploring general techniques that underlie misinformation production. A series of studies show that revealing the techniques of manipulation—including tactics such as polarizing people, using moral-emotional language, relying on fake experts and creating conspiracy theories—can effectively inoculate people against misinformation that they have not seen before, consistent with the notion of attitudinal ‘cross-protection’ [32]. These technique-based inoculations were delivered in the form of games, such as *Bad News*, *Go Viral!, Radicalise* and *Harmony Square*, which simulate a social media environment and pre-emptively expose people to weakened doses of the strategies used in the production of misinformation. These games have shown to improve people's ability to recognize misinformation, boost people's confidence in their own truth-discernment abilities, and reduce self-reported willingness to share misinformation in people's social networks [13,14,29,33–36], effectively encouraging people to actively generate their own misinformation ‘antibodies’ [14,33]. We note here that an effect size of *d* = 0.3 is the lower-bound effect size for a typical inoculation intervention in the field, which forms the basis for a citizen's ability to detect misinformation in the simulation described below. Such general or ‘broad-spectrum’ immunity against future exposure to misinformation through evidence-based interventions ties into research conducted within the sphere of information science, and specifically information behaviour [37]. Researchers in this field have argued for a convergence between research and practice when it comes to designing interventions that seek to improve how people search for and use information, including news sources [38]. For example, previous research into this area has found that an appropriate learning and teaching intervention that incorporates aspects of digital and information literacy can engender ‘pro-active scepticism’, which enhances people's information discernment and enables them to make better news consumption and sharing decisions [39].

Yet, recent reviews have identified one major open question: how to move from the development of a cognitive vaccine to population-level herd immunity [11,16]. Indeed, scholars have argued that the most important yet underdeveloped area of inoculation theory research is how the vaccine can spread from one individual to the next and unfold within a social network [24,40]. It has been hypothesized that cognitive inoculation can spread beyond those who have directly received the ‘vaccine’ through word-of-mouth communication within social networks [40].

At present, little computational research exists on inoculation theory that explores how the spread of inoculation in a social media environment might confer population-level herd immunity (for exceptions see [20,25]). This is a complex question. First, inoculation interventions are known to decay over time [10,34]. Second, misinformation and inoculation techniques may spread socially through bottom-up networks. Given known decay rates and effect sizes of inoculation interventions, what percentage of an online population needs to be inoculated so that misinformation no longer has a chance to persuade people? What are the characteristics of the spread of misinformation in an online network when people can share the psychological inoculation they receive versus when they cannot? What role do factors play such as prior beliefs and trust in the message? To what extent are these findings dependent on the structure of the social network? And is it more efficient to inoculate a large percentage of the population up front as part of an inoculation campaign or to let the vaccine diffuse organically throughout a population over time? The current research seeks to provide preliminary answers to these important yet unanswered questions. Because these questions rely on a complex and dynamic system of interacting agents [25], we use an agent-based model (ABM) in the current study to explore these questions.

### 1.2. Agent-based models

Due to dynamic interactions between people over time, social media platforms can be described as *complex.* A complex system is computationally irreducible [41]*.* This means that the evolution of a macro state that emerges from a system cannot be fully expressed in equations, even if a low-level understanding exists. Complex problems cannot be described or predicted by ‘multiplying-up’ each component in isolation [42]. ABMs can be used to capture dynamics and emergent properties of complex systems, explore the consequences of cognitive and social psychological models and simulate the impact of policy ([18], see also [43], for a discussion of ABMs and policy).

ABMs are computer-simulated multi-agent systems that describe the behaviour of and interactions between individual agents operating in synthetic environments [44,45]. ABMs have been used in economics [46], the social sciences ([47,48], see [49] for an overview) and ecology [50]. They are interactive systems [51] with self-organizing capacities [52]. ABMs consist of three general elements: *agents* (the individual actors within the model), *environment* (the synthetic world in which the agents (inter)act) and *interactions* (connections between agents within the model). This makes them ideal to explore the above questions, as they can represent cognitive features (e.g. belief revision and impact of inoculation), social features (e.g. passing information or inoculation techniques to other agents in their network) and interventions (e.g. different inoculation training interventions).

ABMs are particularly relevant to study how misinformation and inoculation methods may spread in social networks, as this relies on individual, social and structural factors. On the individual and cognitive level, agents revise their beliefs when encountering new information, on the social level, they may prune their networks and potentially share inoculation techniques with their friends, and on the structural level, broadcasters and the structure of the network itself impacts citizen beliefs. To test how psychological inoculations may impact these complex and dynamic information systems, we integrate inoculation theory within an ABM. Previous work has used ABMs to explore belief diffusion [53], cascading effects [19], echo-chamber formation [17,51], micro-targeting [54,55], how climate change scepticism can spread in social networks [56] and emergent polarization [57].

## 2. Model description, assumptions and settings^1^

We populate the model with two agent types: citizens and broadcasters. Citizens are exposed to information from both broadcasters and peers. Citizens seek to communicate their beliefs honestly and to the best of their ability. We note here that communication is captured via a mechanism of public ‘posting’ information, to which one's peers may or may not attend. Comparatively, broadcasters may share information for various reasons, including purposeful biasing or misinforming, as well as for accuracy or truthfulness. Citizens are randomly allocated across a two-dimensional space where they can communicate and form linked networks with each other. These networks are formed by citizens as they search for information over the course of the simulation run, thus localized networks are constructed organically in response to their evolving internal and external information contexts, rather than assumed as pre-existing structures. These localized networks are assessed when determining echo-chamber effects.

Citizens evaluate new information in two ways: first, they assess the subjective perception of the credibility of the source (see Method/technical appendix) and dismiss information from sources that are deemed not to be credible. Second—and novel to this work—they assess the information for signs of misinformation. In line with work on inoculation against misinformation techniques [13–15], communicated information carries with it misinformation ‘cues’ (given a numerical value in the model, in the real world these might be, inflammatory language, conspiratorial reasoning, or logical fallacies such as ad hominem attacks; see [35]—a full discussion of the types of misinformation and persuasion cues is beyond the scope of the current paper, but has been studied in areas such as cognitive psychology [58], social psychology [59] and rhetoric [60]) that are correlated with the likelihood of information being false or misrepresentative. In line with inoculation theory, the citizens will reject information they deem to be misleading (i.e. misinformation) on the basis of detected accompanying ‘cues’. The likelihood of a citizen detecting these cues is a function of the number of cues attached to the communication, the baseline sensitivity of the citizen to misinformation cues, and the citizen's exposure to *inoculation training*. In our model, this process is informed by empirical work on inoculation training (e.g. [13,35]), and the parameters of training are either calibrated to existing empirical data (e.g. training efficacy) or manipulated between simulations to explore our hypotheses (e.g. how many citizens to inoculate in order to achieve ‘herd immunity’ against misinformation, how many times they require the training, and over what time period?). The model leaves open the option for false positives, such that citizens may misidentify well-intentioned communications as misinformation (e.g. if they are ‘over-sensitive’ to cues), reflecting a real-world possibility of inoculation training initiatives [13].

If citizens do not reject the communicated information (based on the above), they update their beliefs in a Bayesian manner in light of the information they have received, using the perceived credibility of the source, and the coherence of this information with their current beliefs, according to the Bayesian Source credibility model (see Method/technical appendix). Along with the update of their beliefs (i.e. *what they believe*), citizens similarly update their confidence in their current beliefs (i.e. *to what degree do they believe they are correct in their current belief*). We note here that our definition of equally ‘rational’ agents is based on agents' use of this inaccuracy minimizing belief-updating Bayesian model (e.g. [61]), and their related search criteria to prefer sources deemed credible (again representing inaccuracy minimization motivations)—cognitive machinery implemented and used across all citizens equally.

Citizens then decide whether to share information with their peers, and if so, whether to simply pass on the information they were exposed to (e.g. sharing the link to the same news article), or their own generated opinion (which is a product of all pieces of information the citizen has encountered). This decision-making process is a function of their perceived credibility of the source of the information they have assessed, and their own confidence in their belief, wherein sources the citizen deems highly credible are more likely to be directly shared, and higher confidence citizens are more likely to want to pass on their own opinions (see Method/technical appendix). As a result, information shared (or ‘posted’) by citizens will be found and integrated by a number of fellow citizens (i.e. a successful ‘communication’) within a given time point as a function of how popular (or acceptable) the content of their shared information may be. More precisely, on the one hand less acceptable items of shared information will have a smaller potential audience of searching citizens, but among those searchers will be found by a higher percentage of that audience (i.e. in the extreme, there is a smaller audience, but fewer public declarations for that audience to find). On the other hand, a more broadly acceptable item of information has the potential to reach a far larger audience, but that audience will have a far larger number of sharing citizens to choose between (i.e. in the mainstream, someone sharing information will have a smaller share of a larger potential audience).

Finally, having decided to share (or not) a piece of information with their peers, citizens lastly seek to prune their local networks, such that any peers who are actively sharing information that runs contrary to the updated beliefs of this citizen (i.e. information that is now deemed not credible) are now severed from the immediate network of this citizen.

Thus, within each simulation a ‘round’ consists of each citizen receiving information, evaluating that information, updating their beliefs in light of this new information (if it passes evaluation), deciding on this basis whether to share this information (or their own, updated beliefs) with others, and pruning their networks in light of their updated beliefs. The simulation continues until either a predetermined end point has been reached (in the current simulations, this is set at 25 ‘rounds’), or until all citizens are confident that they are correct in their individual beliefs.

Within this context, we manipulate two central variables. First, the influence of inoculation training at the start of a round, and second, the influence of broadcasters sharing (mis)information at the start of a round.

### 2.1. Operationalizing inoculation in the agent-based model

Informed by recent empirical work, we deploy different forms of inoculation training aimed to reduce susceptibility to misinformation. Inoculation training takes place in the model at the start of a simulation ‘round’, where a randomly selected number of citizens (this number is manipulated) receive training. The number of participants targeted for training is manipulated (*Inoc*_Num_), along with how often training occurs during the simulation (*Inoc*_Freq_).

This training raises a citizen's sensitivity to misinformation cues by a fixed amount (*Inoc*_Effect_ = 0.3, based on effect sizes of anti-misinformation inoculation interventions reported in previous inoculation research; see [13,14]). This effect decays linearly for each time point after training, such that decay was fixed at 0.075 per time point after an initial 2 time point period for the current simulations (this period and subsequent decay is informed by results reported by [15]). Lastly, we manipulate whether those citizens that have received training will share their training with peers (fixed at 5% for current simulations, see [13]). Peer-to-peer sharing of the inoculation training (if enabled) also occurs at the start of a simulation round (i.e. prior to information communication/sharing functions).

#### 2.1.1. Broadcasts

Broadcasters within the model operate by disseminating their ‘belief’ (i.e. (mis)information) to all citizens within the network in the communication phase of a simulation round. How often a broadcaster communicates (*BC*_Freq_; i.e. how often does a round include a broadcaster communicating, rather than just peer-to-peer sharing) is manipulated. In the current simulations, we implement two types of broadcaster strategies (*BC*_Strat_): an *honest* broadcaster that communicates a belief value corresponding to the ground truth (i.e. 0.5 in the current simulations), or a *biased* broadcaster that communicates a belief value deviating from the ground truth (i.e. 0.75). Aside from the communicated value, these two broadcaster strategies operate in the same manner. When both broadcasters are present in the simulation, they take turns to communicate with the citizenry, such that only one broadcaster communicates during any one simulation round.

When a broadcaster communicates, along with the belief value, it also generates accompanying misinformation cues (represented along a single dimension between 0 and 1). These cues are determined based on the degree to which the intended communication belief value deviates from the broadcaster's understanding of the typical (i.e. average) audience belief value, such that communications that deviate further from audience expectations are correlated with increasing misinformation cue deployment (see Method/technical appendix for details). As mentioned in the above, citizens use these cues, along with the perceived credibility of the broadcaster, to evaluate the broadcasted information, either dismissing it, or taking that information forward to update their beliefs. If a citizen decides to directly share a broadcaster's content, then the misinformation cues attached to the original broadcast are preserved when shared with peers.

In summary, each citizen starts off with a prior belief about the world (*µ_own_*), based on independent samples from a parent distribution, a baseline confidence in their belief (*P*(*H|E*), fixed to 0.05 for these simulations), and a baseline sensitivity to misinformation cues (*Base_MCS_*). In this way, citizens are heterogeneous in their beliefs and their baseline sensitivity to misinformation, but homogeneous in their (lack of) confidence at the beginning of the simulation. In the current model and simulation runs, misinformation is only shared by broadcasters. Of course, in actual social networks, bad faith actors who willingly distribute mis- and disinformation is a significant challenge [62]. Extending malicious intentions to the citizenry would represent an additional challenge. As such, in the current simulations, citizens rationally share information they think is most credible. Future work should extend the challenge of the spread of misinformation in social networks to also include bottom-up contributions from bad faith actors, monetized incentives for spreading disinformation, and the spread of malinformation. However, these dynamics go beyond the scope of the current paper.

#### 2.1.2. Manipulations

##### 2.1.2.1. Inoculation training manipulations

To determine the potential interplay between complex system dynamics and inoculation training, we initially deploy three conditions to test inoculation training effects. First, we have a baseline *No Training* condition, where there is no training conducted throughout the simulation (i.e. *Inoc_Freq_* = 0; *Inoc_Num_* = 0). This condition serves as a baseline comparison for the other training conditions. Second, we have an *Upfront* training condition, where a one-off percentage of the citizenry receives the inoculation training at the beginning (in this case, 60% of citizens, i.e. *Inoc_Freq_* = 1; *Inoc_Num_* = 60%). Third, we contrast this with a *Staggered* training condition, where the same percentage of citizenry is trained, but spread over multiple, smaller training sessions (in this case, three sessions of training 20% of citizens, with one occurring every five time points—i.e. initial, 5th and 10th ‘rounds’; *Inoc_Freq_* = 3; *Inoc_Num_* = 20%). These conditions allow us to investigate the efficacy of training when considering the temporal dynamics in play within the social network (e.g. echo-chamber formation over time, preventing further intervention).

##### 2.1.2.2. Broadcaster manipulations

We manipulate three broadcaster scenarios. First, when there is only a single, *honest* broadcaster (i.e. *BC_Strat_* = *Honest*, where the broadcasted value = 0.5), communicating twice every three time points. Second, when there is only a single, *biased* broadcaster (i.e. *BC_Strat_* = *Biased*, where the broadcasted value = 0.75), communicating twice every three time points (i.e. *BC_Freq_* = 2/3). Third, when there is both an *honest* and a *dishonest* broadcaster competing, with each communicating once every three time points (staggered, such that each broadcaster communicates on a separate time point, with a third time point left for peer-to-peer communication only). Thus, the three broadcaster conditions are *Honest*, *Biased* and *Competing*.

##### 2.1.2.3. Citizen manipulations

We manipulate two citizen scenarios. To understand the potential efficacy of misinformation (and training to inoculate against it), it is important to understand the impact of how sensitive citizens are to misinformation at a baseline level. Accordingly, we manipulate three levels of baseline citizen misinformation cue sensitivity, *Low* (*Base_MCS_* = 0.05), *Medium* (*Base_MCS_* = 0.2), and *High* (*Base_MCS_* = 0.35). These settings cover a broad range of hypothetical values, covering sensitivity values informed by empirical work [13–15].

#### 2.1.3. Dependent variables

We are interested in three primary dependent variables to assess the impact of inoculation training. Each is measured over the entire time period of the simulation (i.e. 25 time points).

##### 2.1.3.1. The distribution of citizens' beliefs

The belief value of each citizen (*µ_own_*) is recorded within a distribution, allowing us to determine the dispersion or clustering of beliefs across the entire citizen population. This is important to inform understandings of global versus local patterns of behaviour (e.g. degrees of network coalescence).

##### 2.1.3.2. The purity of citizens’ local networks

This measure takes the amount of agreement within the local (i.e. direct) network of a citizen, using the squared difference in belief values between a citizen and their directly connected peers. Inverting this value (1 – value), 1 corresponds to complete agreement between a citizen and their directly connected peers, while 0 corresponds to maximal disagreement. The mean purity across citizens is then used as an indicator of echo-chamber formation (i.e. epistemic purity is a key indicator of echo chambers).

##### 2.1.3.3. The numbers of misinformation rejections and false positive rejections

As the simulation progresses, we record the number of times citizens, having received a communication that has passed credibility evaluations, decide to reject that communication on the basis of misinformation cues. We additionally split these rejections on the basis of their proximity to a ground truth (for now, set as ±5% from the ground truth, 0.5), wherein those sufficiently close to the ground are considered a false positive rejection (i.e. an erroneous rejection of the ‘truth’), while communications with a value outside of this proximity are considered correct rejections of misinformation.

Further, we are also interested in three secondary dependent variables to more comprehensively check inoculation efficacy, and better understand the context of citizen beliefs. These are:

##### 2.1.3.4. The percentage of citizens who have received the training

To monitor the effects of inoculation training initiatives, we record the percentage of the population of citizens that have received the training—irrespective of whether this inoculation has been provided to a citizen externally, or shared laterally between citizens.

##### 2.1.3.5. The levels of misinformation cue sensitivity among citizens

Across the simulation, we measure the average misinformation cue sensitivity of citizens. This allows us to investigate how levels of cue sensitivity are correlated with changes in the distribution of beliefs among citizens (e.g. in light of attempted dishonest broadcasts). It is further important to keep track of this sensitivity, given its complexity: citizens have a baseline sensitivity, modified by training, and the effect of that training decays over time (but may be refreshed by further training).

##### 2.1.3.6. The confidence citizens have in their beliefs

At each time point, we measure the confidence citizens have in their current belief (*P*(*H|E*)) being the truth. Confidence is updated according to the Bayesian source credibility model (see Method/technical appendix) each time a citizen has received information that has passed their evaluation checks. As the degree to which citizens will engage in further search and updating is dictated by their level of confidence (i.e. a citizen confident they are correct, will not search out further information: see Method/technical appendix), it is useful to measure how quickly confidence builds to determine factors like receptiveness to new information.

For all dependent variables, the simulation approach allows us to look at how these effects unfold over time. In our simulations, each parameter permutation was run 100 times, with the results averaged for plotting. The model was implemented in *NetLogo* v. 6.0.4 [63], and simulations were conducted using the RNetLogo package in R [64].

## 3. Results

Simulations were conducted using a broad range of parameter values, and a range of dependent variables were measured for each permutation. For the sake of brevity, not all results are reported here in the main text. The remaining results not reported in the main text are reported in the electronic supplementary materials (available at https://osf.io/vjk64/). For the sake of clarity, we present here:

Findings regarding the levels of misinformation cue sensitivity among the citizenry, to investigate the *direct impact* of inoculations on misinformation sensitivity; the corroborative percentage of the network that has received training is reported in the appendix. To follow this through to the *evaluative process impact*, we investigate a subset of the misinformation rejection and false positive rejection data (‘medium’ baseline citizen sensitivity to misinformation); with the remaining conditions (‘low’ and ‘high’ baseline citizen sensitivity to misinformation) detailed in the electronic supplementary materials.

Next, to investigate the impact of evaluative process changes on the beliefs of citizens, we investigate the same subset of the belief distribution data (with remaining baseline citizen sensitivity to misinformation conditions also reported in the electronic supplementary materials).

Given the importance of temporal sensitivity to the belief formation process (as found in previous work, e.g. [17]), confidence citizens have in their beliefs over time is also investigated here. The related purity of citizens local networks, though briefly explained in the main text, is more substantially reported in the electronic supplementary materials, appendix.

### 3.1. The direct impact of psychological inoculations (manipulation check)

**Figure 1.**
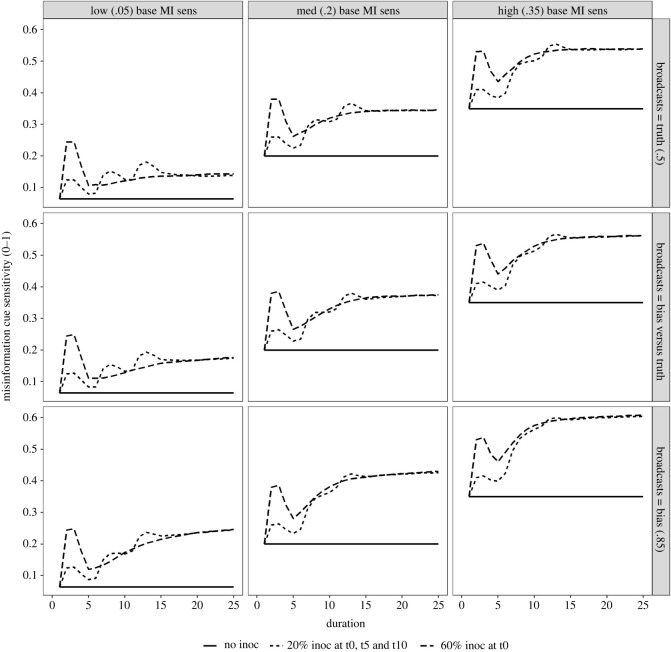
Citizen misinformation cue sensitivity (0–1) over time, with line-types reflecting the three inoculation training conditions. Broadcaster conditions are shown on facet rows, and baseline citizen misinformation cue sensitivity conditions are shown on facet columns.

To determine the direct impact of inoculations on the intermediary psychological component (i.e. citizen sensitivity to misinformation), we investigate the misinformation cue sensitivity of citizens over the course of the simulation. This serves in part as a manipulation check and also explores the temporal dynamics of a citizenry that can also share inoculation training with one another. Figure 1 shows that when there is no inoculation training (solid lines, figure 1), the baseline sensitivity condition (columns, figure 1) dictates the constant level of misinformation cue sensitivity throughout the simulation.

Both training regimes (60% at time point 1, versus 20% over three time points—1, 5 and 10) yield similar levels of misinformation cue sensitivity—regardless of baseline sensitivity or broadcaster condition, their pathways to reaching this ‘saturation point’ differ. This ‘saturation point’ is affected by the baseline misinformation cue sensitivity and the broadcaster condition. More precisely, the higher the baseline sensitivity, the more effective (i.e. the larger the increase) the training is on the final ‘saturation point’, and the more predominant a biased broadcaster is (i.e. from no biased broadcaster, through competing with a truthful broadcaster, to there only being a biased broadcaster; top to bottom row of figure 1), the higher still this ‘saturation point’ reaches. Citizen activity is the likely reason for this. When citizens have a higher level of baseline misinformation cue sensitivity, it produces a higher intention rate of re-sharing the inoculation (citizens who are more attuned already to detecting misinformation are also more intent upon dissemination of assistive information). Second, when citizens are faced with biased broadcasters, there are longer periods of deliberation on average among them (as there is more belief-updating required to move the priors to the biased posterior). This longer period of active engagement among more of the citizenry allows for more of the ‘mass’ of citizens to share inoculation training later into the simulation.

Finally, in comparing the two inoculation regimes directly, differences are found earlier in the simulation timeline. The misinformation cue benefit of training provides an initial benefit (which is more pronounced in the single 60% training condition), but then dips as those initially trained start to experience a decay that competes against the rate of training sharing. As the mass of willing inoculation sharers grows (faster when baseline misinformation cue sensitivity raises the rate of sharing), a critical mass of sharers builds to counteract decay rates. Eventually the dynamic then shifts to favour a new, ‘trained’ equilibrium. Although this process is ‘smoother’ when training is staggered, the larger spike of sensitivity at an earlier, potentially critical, time period (even accounting for the dip from this spike, which is still higher than the sensitivity of the staggered regime at the same point in time) plays an important role in the knock-on effects to the evaluative and belief formation processes of citizens, as explained below.

### 3.2. The effect of sensitivity changes on the evaluative process of citizens

**Figure 2.**
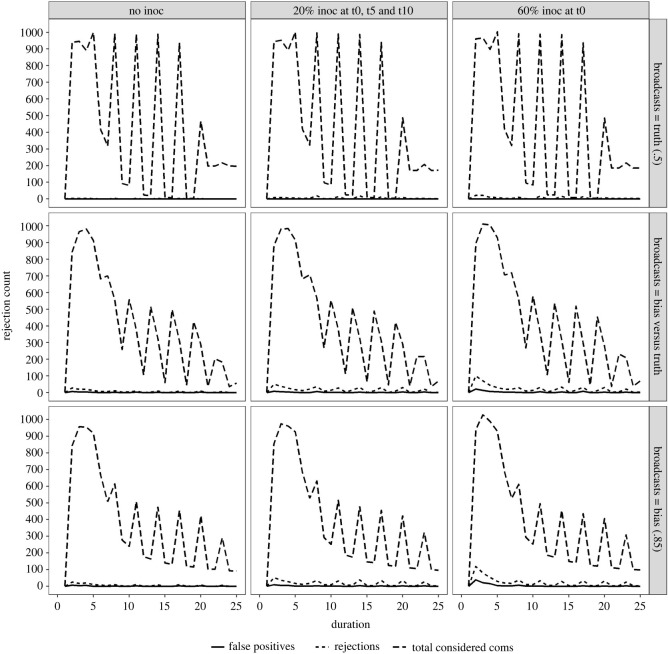
Communications, correct rejections and false positive rejections over time. Medium (0.2) baseline misinformation cue sensitivity condition. Inoculation training conditions are shown on facet columns, and broadcaster conditions are shown on facet rows.

To determine the carry-through of inoculation effects onto the intermediary evaluative mechanisms, we turn next to the rates of misinformation rejection among citizens. Importantly, we contextualize these rejections by plotting them against both the overall amount of communication occurring at a given time point and the number of false positive rejections (i.e. rejections of information that lies within ±5% of the ‘true’ value), illustrated in figure 2 below.

Broadly, we observe the general trend of reduced amounts of communication as simulations progress (dashed lines, figure 2), reflecting the belief consolidation process as agents update their beliefs. The spikes in these trends reflect broadcaster dissemination to agents, which affects all those who find the information credible. This is why, when the broadcaster is truthful (top row, figure 2), broadcasts continue to reach the majority that has consolidated around this truthful value (an effect borne out in belief distribution data, see next section). However, when biased broadcasters are included, broadcaster reach becomes gradually less effective, reflecting the consolidation of beliefs among those rejecting the biased content. Lastly, the ‘dips’ between peaks in the total amount of communications reflect how much peers are searching and accepting each other's shared information between broadcasts with rapidly decreasing trends again showing the temporal sensitivity of the belief formation process.

Turning to the effects of inoculation training conditions (columns, figure 2), we note that although these do not affect overall amounts of communication, they do affect the amount of misinformation rejections (dotted lines, figure 2) during the early stages of the simulation, when communication and belief formation are most prolific. Importantly, rejections occur at this early stage the more biased broadcasters have command of information dissemination (i.e. least rejections when truthful broadcasters have sole command (top row, figure 2); more when a biased broadcaster is competing with a truthful broadcaster (middle row, figure 2); and more still when only a biased broadcaster has sole command (bottom row, figure 2). This suggests inoculation training is targeting its intended purpose (i.e. increasing the rates of misinformation rejection), without affecting more truthful information dissemination. Such an effect is further borne out by the rates of false positive rejections (solid lines, figure 2), which remain low throughout all conditions, albeit fractionally higher when large numbers of rejections are occurring (e.g. biased broadcaster ×60% initial inoculation condition).

This leads to the final effect discussed here^2^: given the identified temporal sensitivity of the belief formation process among citizens in the network, we note the higher rates of misinformation rejection in the early critical period of the simulation, when a 60% one-off initial inoculation training regime is used (versus a more spread-out set of inoculations over 15 time points; right-hand versus middle column, figure 2).

### 3.3. The impact on citizens' belief (formation)

We turn next to measures of the belief formation process itself—the distribution of beliefs across the population of citizens, and the confidence those citizens have in their beliefs. Having explored both the direct impact of inoculations on misinformation sensitivity, and the subsequent impact on the evaluative processes of citizens (i.e. their capacity to identify and reject misinformation), it is necessary to understand how these changes affect the belief landscape itself: what do citizens end up believing, and how swiftly do they become confident that their belief is correct?

**Figure 3.**
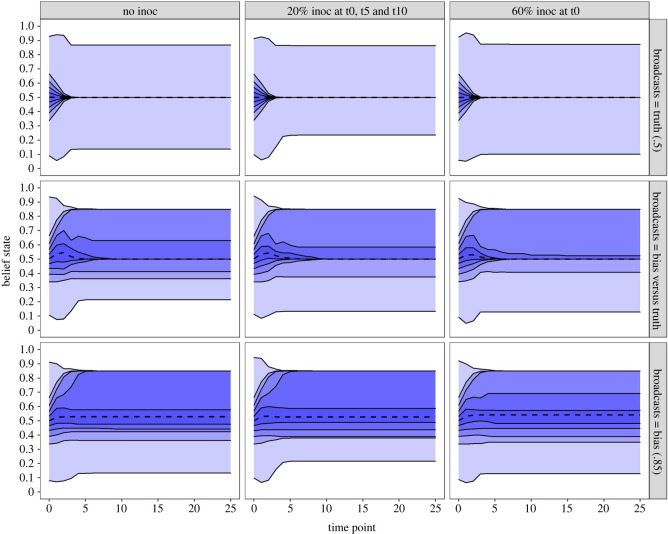
Distribution of citizen beliefs (*μ*_*own*_) over time. Medium (0.2) baseline misinformation cue sensitivity condition. Dashed line reflects median belief, quantiles reflect ±10% bands outward from median. Inoculation training conditions are shown on facet columns, and broadcaster conditions are shown on facet rows.

To address the first question, figure 3 illustrates the distribution of citizens’ beliefs (*μ*_*own*_) over time, separated by inoculation training condition (columns) and broadcaster conditions (rows). First, when broadcasters are truthful (top row, figure 3), we find that inoculation training does not impede convergence of beliefs on this truthful value—corroborating our findings of low levels of communication rejection when truthful broadcasters are disseminating information. Second, when biased broadcasters are present (middle and bottom rows, figure 3), the ‘mass’ of citizen beliefs is less impacted by the pull of these biased broadcasters. More precisely, the quantiles indicate that fewer citizens are persuaded toward the biased broadcasters' communicated value of 0.85, when inoculation training is in place. However, we note that this effect is more pronounced when the inoculation is a single, early inoculation of 60% (e.g. bottom-right facet, figure 3) than when that 60% is spread over a gradual time period (three sessions of 20%, see bottom-middle facet, figure 3). This further corroborates the demonstration of the importance of time sensitivity (i.e. temporal dependence) when considering misinformation prevention across online networks where peer-reinforcement exists.

Although figure 3 illustrates citizen belief distributions over time when baseline misinformation sensitivity is at a ‘medium’ value (0.2), we point the reader to the electronic supplementary materials (available at https://osf.io/vjk64/), where high and low baseline misinformation sensitivity conditions are also shown. There, we show that the higher the baseline misinformation cue sensitivity, the more pronounced the above inoculation training benefits become for citizens' resistance to biased broadcasters.

**Figure 4.**
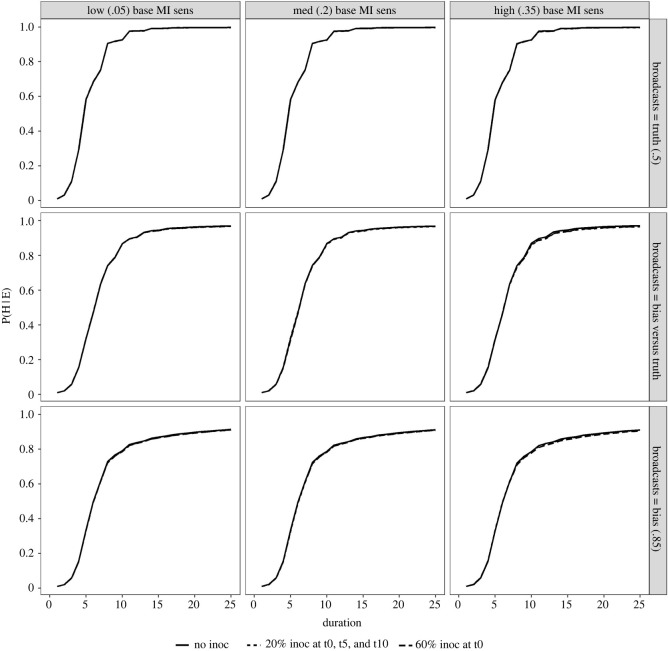
Confidence citizens have that their beliefs are correct (P(H | E)) over time, with line-types reflecting the three inoculation training conditions. Broadcaster conditions are shown on facet rows, and baseline citizen misinformation cue sensitivity conditions are shown on facet columns.

Finally, we turn to the confidence citizens have about their beliefs being true (P(H|E)), plotted over time in figure 4. The s-curve shape of the confidence lines illustrates both the accrual process of confidence acquisition, and also the temporal sensitivity of the belief formation process—as citizens become less open to new information, the more confident they are that their own belief is correct. This corroborates previous findings of confidence acquisition in belief formation across social networks [17].

As we can see from the overlapping lines (solid, dashed and dotted, figure 4), the inoculation training condition does not affect the rate at which citizens become confident about their belief. Put another way, inoculations may impact *what we believe* (see figure 3), but not *how quickly we acquire a belief.* This is further underlined by the fact that baseline misinformation cue sensitivity (columns, figure 4) does not affect the rate of confidence gain. Taken together these findings suggest that the in rich networks (i.e. where plenty of information is available from potential peers, not just broadcasters), information that passes citizens evaluation process can always be found—resulting in an inevitability of the belief formation process. Of course, further work should investigate the potential mathematical relationship here between available sample size and evaluation thresholds.

Lastly, we find lower degrees of confidence (and shallower slopes of confidence accrual) when citizens are faced with a biased broadcaster (bottom row, figure 4), especially when contrasted with the impact of a truthful broadcaster that reinforces the population prior (top row, figure 4). This effect reflects the uncertainty provoked by persuasive attempts that lie further outside prior expectation, especially when coupled with the contradicting information shared by like-minded peers.

## 4. Discussion and conclusion

In this study, we conducted a first exploration of inoculation theory within the context of agent-based modelling (ABM), using inoculation efficacy benchmarks established in previous inoculation research. We asked whether inoculation training (e.g. by playing ‘fake news games’) (i) can reduce misinformation susceptibility and belief polarization at a population level (rather than at an individual level, as has been explored in previous research); (ii) can do so without inadvertently backfiring by making people oversensitive to reliable information (i.e. false positive rejections of information); (iii) can remain effective over time.

First, we find that both ‘upfront’ (inoculating approximately 60% of the population simultaneously) and ‘staggered’ (inoculating smaller groups within the population at different points in time) approach increase misinformation cue sensitivity (i.e. how good people are at picking up on misinformation cues in news content) at the population level, with both approaches eventually reaching an equilibrium of misinformation cue sensitivity that is significantly higher than if no inoculation treatment were administered. Our findings thus offer initial support for the feasibility of leveraging inoculation theory to achieve psychological ‘herd immunity’ against misinformation. In the simulations, we operate with 60% inoculated citizens, which is probably optimistic, but the simulations provide proof in principle that herd immunity is achievable through inoculation for the information scenario we describe in the model. Furthermore, we find that the number of correct misinformation rejections (i.e. a citizen choosing to ignore a piece of misinformation rather than allowing it to influence their beliefs) increases significantly post-inoculation, and far outpaces the number of false positives (i.e. wrongful rejections of reliable information as misinformation), indicating that the risk of inadvertently increasing broad-level scepticism of ‘real news’ through inoculation interventions is likely to be low.

Second, in terms of belief polarization, we find that inoculation trainings can impact how much a population is affected by biased broadcasters. In other words, while the inoculation training has no effect on people's perceptions of reliable broadcasters, the ‘pull’ of biased broadcasters (who are more likely to use misinformation cues as a persuasion mechanism) is less impactful when a population is inoculated. This effect is more pronounced when citizens' baseline level of misinformation cue sensitivity is higher. This has potential implications for longer-term strategies aimed at countering misinformation at a societal level: educational initiatives aimed at raising baseline misinformation cue sensitivity may play an enhancing role in increasing the efficacy of later inoculation interventions. In other words, the more familiar a population is with the tell-tale signs of misinformation, the more impactful inoculation interventions are likely to be.

Third, we find that the impact of inoculation trainings in terms of the number of misinformation rejections overwhelmingly occurs early on, i.e. before people's beliefs about a particular topic have crystallized. This finding has implications for inoculation training design, given the temporal dynamics of belief formation across a network, suggesting it may be better to front-load trainings to pre-empt misinformation initiatives before the network reinforces the consolidation of (misinformed) beliefs. In other words, inoculation interventions are likely to be more effective when administered before or immediately after a persuasive attack or misinformation campaign.

Our study design has several important limitations. First of all, agent-based models (ABMs) are limited in their capacity to model real-world human behaviour, and ours relies on relatively simple assumptions about news consumption, sharing behaviour, belief polarization and the effects of misinformation exposure. Second, our model was not able to take into account peer-to-peer instigation of wilful sharing misinformation (we built our model so that only broadcasters wilfully share misinformation, not agents). Third, we were unable to account for complex dynamics surrounding individual broadcasters' or narratives’ persuasiveness or the varying effects on different groups within society (as well as potential resistance to being ‘inoculated’, which may reduce the efficacy of the training for some agents and groups). We acknowledge the role that future research can play in behavioural validation (and extension) of the findings laid out in principle here.

Nonetheless, using agent-based modelling to assess the effects of administering inoculation interventions at a population level allows us to draw several (tentative) conclusions: (i) large-scale inoculation programmes can be effective at reducing misinformation susceptibility and the persuasive power of biased broadcasters at scale; (ii) the risk of inadvertently increasing general scepticism of reliable information appears low; (iii) the impact of inoculation interventions is most prominent early on, before people's beliefs have crystallized, which is consistent with predictions from inoculation theory [16]; (iv) inoculation programmes are likely to be more effective when a population's baseline ability to identify misinformation is higher, highlighting the potential benefits of media- and digital literacy education [65] on top of administering inoculation trainings. These findings have important implications for policymakers as well as social media companies: when choosing to inoculate, it is probably better to do so early on (before, during or just after a targeted misinformation attack takes place), and to aim at reaching as large a share of the population as possible.

## 5. Method/technical appendix

To explore the effects of misinformation inoculation training on a human information system, replete with phenomena like emergent echo chambers, we employ an agent-based model. This allows us to outfit a population of agents (e.g. citizens) with relevant cognitive functions (Bayesian belief revision), the capacity for interaction (sharing their beliefs), create broadcasters that disseminate information according to predetermined strategies over time, and deploy inoculation (training) initiatives among citizens.

The model specifically explores whether misinformation inoculation training can assist in reducing the misleading effects of misinformation (e.g. when disseminated from dishonest broadcasters) as information sharing and updating plays out across social networks of citizens. Included within the scope of possible emergent behaviour, via the interactions between citizens, who search, update and prune their (social) information networks (which in turn informs the information context, and subsequent behaviours, of their peers), are phenomena like echo-chamber formation and entrenchment. In the following, we describe how the social network is structured, how broadcasters and citizens are initialized, how they engage with one another, how citizens are ‘inoculated’, and how all of the above influences the evaluation and updating process, as well as subsequent search and pruning behaviours.

### 5.1. Model set-up

Citizens are part of a randomly connected social network. To initialize the network, 1000 agents (classed as citizens) are placed on a 100 × 100 spatial grid in random positions. Upon initialization, each citizen generates the necessary variable values for operating within the simulation, these are:

The baseline sensitivity a citizen has to detect misinformation cues (*Base_MCS_*), drawn from a truncated normal distribution (0–1) with standard deviation 0.05, and mean manipulated across three permutations: *Low* (*Base_MCS_* = 0.05), *Medium* (*Base_MCS_* = 0.2) and *High* (*Base_MCS_* = 0.35).

The credibility halo that dictates the degree of openness (in terms of acceptable credibility) citizens have is a fixed value of 1 in the present simulations, but values greater than 1 result in an expanded credibility ‘halo’ around the credibility distribution (which is overlaid on the belief distribution of the agent, explained below).

The initial belief distribution each citizen starts with are generated through a several-stage process. First a ‘raw’ sample (size = 5) is drawn from a truncated normal distribution with a mean of 0.5 (reflecting the ‘true’ state of the world) and standard deviation of 0.25 (truncated between 0 and 1). This ‘raw’ sample is then weighted by two factors: temporal position (how recently was information received) and perceived credibility associated with source. For initialization, the latter is held constant across each sampled information item (5 for all), but when the information starts to arrive from sources during the main simulation, these numbers change (explained below). The temporal weighting is based on multiplying position number (1 = oldest, to 5 = newest, given a ‘raw’ sample size of 5) using an exponent set to a fixed weighting value, in this case 2. More formally:

5.1 $$TempWeight=Position^{2}.$$

Having calculated these two weighting values for each ‘raw’ sample item, the processed belief distribution for each citizen is generated by multiplying the frequency of a ‘raw’ sample item by the two weights. For example, the first (i.e. oldest) raw sample item will appear in the final distribution (1^2^ × 5) 5 times, while the last (i.e. newest) raw sample item will appear in the final distribution (5^2^ × 5) 125 times. This frequency-weighted final distribution allows not only for the generation of a weighted mean belief value that takes into account these two factors (recency and credibility), but also generates an appropriately weighted variance around that mean—something not readily possible via other mathematical methods. The mean of this final distribution thus corresponds to the citizen's current belief (i.e. *µ_own_*). This distribution is then used (and updated) as the simulation progresses, allowing citizens to determine not only their current beliefs, but also their associated distribution of perceived credibility associated with those beliefs (i.e. as information diverges from the central belief, it is deemed increasingly incredible).

Lastly, the confidence the citizen has in their belief being true (i.e. *P*(*H|E*)) is initialized as 0.05 for each citizen. This subsequently updates as the citizen updates their beliefs in light of new information (explained below).

Broadcasters are initialized (based on permutation) as separate agents, with parameters defined by the simulation settings. Specifically, the number of broadcasters (*BC_Num_*), their communicated value (*BC_Strat_*; i.e. their strategy), the frequency of their communication with citizens (*BC_Freq_*), and any necessary staggering of the communication timing (e.g. if multiple broadcasters are active). Broadcasters are not affected by physical position within the simulation, and are thus located in the two-dimensional centre of the simulation space.

At the completion of model set-up, the citizenry has their key variables (e.g. prior beliefs and confidence) initialized, but are yet to form any networks with their peers. Broadcasters are set up to disseminate according to their pre-ordained schedule/strategy, but have not done so yet, and no inoculation training has yet taken place.

### 5.2. Running the model

The model runs across 25 time points. Within each time point, processes occur in the following order (each process is then detailed below):

First, if any inoculation training is scheduled for this time point, it takes place, followed by an opportunity for any trained citizens to share their inoculation training with their peers.

Second, if any broadcasts are scheduled for this time point, those broadcasters ready their broadcasts and disseminate them to all citizens. Citizens will evaluate the broadcast, either accepting or rejecting its contents. If the former is the case, the citizen will update their beliefs in light of this new information, and decide whether they wish to publicly share (i) the original broadcast, (ii) their own (updated) opinion, or (iii) nothing at all.

Third, any citizens that have not accepted broadcasted content (either because no broadcast occurred this time point, or the citizen rejected the broadcast) will search for any publicly declared information shared by their directly connected peers (i.e. their immediate social network). Shared information that passes the citizen's evaluation is then (in the same way as broadcasted content) used to update the citizen's beliefs, and the citizen decides whether to publicly share in the same manner.

Finally, all citizens determine whether, and to what degree, they wish to prune their network links to other agents, in light of the new changes to their own (and their peers) beliefs.

Consequently, we can divide up the requisite functions into seven categories: inoculation training, broadcasting, search, information evaluation, belief updating, information sharing and pruning.

#### 5.2.1. Inoculation training

The inoculation training function is run at the beginning of each time point, and works through three stages:

First, any citizens that have received training in previous time points determine whether they will share their inoculation training with their (directly connected network) peers. This decision rule is based on a likelihood defined by

5.2 $$PeerShareProb\;*\;\left(\frac{MCS_{Own}}{Inoc_{Effect}}\right),$$

where the *PeerShareProb* is set to the expected likelihood of sharing drawn from empirical work (i.e. 5%), multiplied by the ratio of the citizens current misinformation cue sensitivity (which incorporates the effect of the inoculation), to the inoculation effect size itself. In this way, a citizen is more likely to share inoculation training materials, the more they are individually sensitive to misinformation.

If a citizen has decided to share the training materials, all of the peers connected to that citizen receive the training. This means the effect of training is added to the peer's baseline misinformation cue sensitivity and the age-since-training is set to 1. This means citizens receiving training information have the effects of training refreshed if they have previously received training.

Next, all citizens that have received training in previous time points age (1 per time point), and if the age since training exceeds the specified training duration (again drawn from empirical work, and set as 2 time points), then the citizens' misinformation cue sensitivity moves towards their baseline level at a fixed linear decay rate (0.15 sensitivity per time point, again informed by empirical work).

Lastly, if this time point is a determined ‘external’ training round, then a random sample (drawn without replacement within a single time point, but with replacement across time points) of citizens is contacted and provided with the training, adding/refreshing the inoculation effect to the citizens' baseline misinformation cue sensitivity, and setting the age since training back to 1. The size of this sample is manipulated between simulations as a fixed percentage of the total population size (*Inoc_Num_*), as is the frequency of training time points (*Inoc_Freq_*).

#### 5.2.2. Broadcasting

In the same manner as the inoculation training, broadcasts only occur on pre-ordained time points. The number of broadcasters (*BC_Num_*), what those broadcasters choose to communicate (i.e. the belief value; *BC_Strat_*), and when (how often) those broadcasters communicate (*BC_Freq_*) to the citizenry is manipulated as part of the simulation conditions.

Before broadcasting, an activated broadcaster will first generate an associated misinformation cue value (0–1) for their broadcasted message. This cue value is generated via a single sample drawn from a truncated normal distribution (0–1) that takes as its mean

5.3 $$MiCueDistMean=abs(Com_{Own}-Truth_{Own}),$$

where *Com_Own_* is the broadcasters' intended message, and *Truth_Own_* is broadcasters' perception of what the average citizen believes (i.e. the mean *µ_own_* value across the citizen population). In this way, the more the broadcaster is trying to persuade away from the central narrative, the more likely the broadcaster is to rely on misinformation cues to do so (irrespective of the underlying veracity of either their communication, or the public consensus). The standard deviation informing this misinformation cue distribution width for the current simulations is set to 0.2.

Having generated an associated misinformation cue value, the broadcaster (assuming it has been activated to communicate this round), will seek to share their message with all citizens. Those citizens will then evaluate the message based on (i) their perceived credibility of the broadcaster, and (ii) the attached misinformation cue value—described in the Information evaluation section below.

#### 5.2.3. Search

Next, any citizens that have not yet had an opportunity to evaluate new information this round (e.g. there has been no broadcast) conduct a search for any publicly declared information from their fellow citizens. Citizens become increasingly less likely to conduct a search the more confident they are that their current belief is correct (i.e. as *P*(*H|E*) approaches 1).

To conduct a search, citizens first gather the subset of other citizens that are within search range (for the purposes of this manuscript, the search range encompasses all possible social network users, see [17] for a manipulation of this factor), and are actively/publicly sharing information (which may either be their own opinion or simply sharing information they themselves have received). Of this selected subset of citizens, the searcher will determine whether any meet the credibility requirements (i.e. if any other citizens are perceived as credible, given the information they are publicly declaring). This credibility check is performed by determining if the information declared by the citizen (*X*), when contrasted against the credibility distribution of the searching citizen (which is an overlay of the searching citizen's belief distribution), corresponds to a perceived credibility value (the y axis value of that point along the searching citizen's distribution x axis, of height relative to the distribution peak). This height is then compared with the determined credibility cut-off (here set to 0.5, meaning beliefs perceived as less than 0.5 are deemed non-credible), as follows:

5.4 $$\frac{(1/(\sigma_{SC}*\;\sqrt{2\pi }))*\;e^{-({\left(X-\;\mu_{SC}\right)}^{2}/2\sigma_{SC}^{2})}}{SC_{Val}}+CredBonus>SC_{Cutoff},$$

where *SC* is the source credibility distribution of the searching citizen, *X* is the assessed (declared) information, and *SC_Val_* is the peak height of the SC distribution. For citizens evaluating peers, there is no supplementary/independent credibility taken into account (i.e. *CredBonus* = 0). The *SC_Val_* (i.e. peak of the citizens credibility distribution) used to calculate the relative height of the credibility value associated with the information value is calculated as illustrated in equation (5.5) below

5.5 $$SC_{Val}=\frac{1}{\sigma_{SC}*\;\sqrt{2\pi }}.$$

Consequently, if there are any acceptable declaring citizens (i.e. citizens deemed credible by the searcher), then the most recently declaring citizen is passed forward for further information evaluation (i.e. misinformation cue checking). The searching citizen continues through this subset of declaring agents from most recently declaring backwards through time (reflecting the way social media algorithms favour most-recent first posting), until the searcher either finds an acceptable piece of information, or runs out of acceptable declaring citizens. If an acceptable citizen is found (i.e. one who passes the full information evaluation), then a link is forged between the searcher and declarer, representing the immediate peer connection.

#### 5.2.4. Information evaluation

When citizens are exposed to information, it is evaluated in two ways. First, the information is assessed in terms of perceived credibility. When information is searched for by a citizen, this stage of evaluation occurs within the search process (described above). When information has been received from a broadcaster, the same evaluative process (using equation (5.4) above) is conducted, with rejection (and thus no further evaluation) occurring if the perceived credibility of the message source is calculated as below the predetermined cut-off (0.5). However, when it is a broadcaster being evaluated, the credibility calculation of the message also includes an added term for an independent (i.e. not message dependent) credibility ‘bonus’. This corresponds to the potential for broadcasters being deemed credible (or not) to some degree irrespective of the content they are broadcasting. In the present simulation, this broadcaster credibility ‘bonus’ is set to +0.3. Thus, citizens will be more accepting of deviating messages when they stem from broadcasters, as the blanket ‘bonus’ credibility assigned to them by citizens means less credibility derived from message-compatibility is needed to pass the credibility minimums of the citizen.

If information has passed this credibility check, the citizen then evaluates it for possible misinformation. To do this, the citizen compares the misinformation cues attached to that information (a value between 0 and 1), with a sampled distribution value

5.6 $$(1-miValue)\leq TNorm(MCS_{Own},0.25,0,1),$$

such that if the inverse of the misinformation cue value (*miValue*) is less than or equal to the sampled misinformation cue assessment—drawn from a truncated (0–1) normal distribution with a mean equal to the citizens current misinformation cue sensitivity value (and standard deviation = 0.25)—then the information is considered misinformation and rejected. Otherwise, it is accepted as valid/true. In this way, the higher the misinformation cue value, the more likely it is to be rejected, but also the higher the misinformation cue sensitivity of the citizen, the more likely they are to make rejection decisions—irrespective of whether that information is misinformation, or not (representing the possibility of false positives).

For the purposes of categorizing these correct/incorrect rejections, a classifier decision is added for simulation recording purposes. This records the total number of rejections at each time point, but also those rejections made for information that in fact lies within ±5% of the ‘ground truth’ value used in the simulations (0.5)—reflecting an approximation of a false positive rejection.

#### 5.2.5. Belief updating

To update their beliefs, citizens first determine the related credibility values associated with the information they have received this round, using the source credibility height calculation described above (left-hand side of equation (5.4)), and any independent credibility ‘bonus’ is added (i.e. if the information is sourced from a broadcaster whom the citizen deems independently credible of information communicated). It should be noted that if the citizen failed to find any relevant information, then this calculated credibility value is set to 0.01, reflecting the lack of credibility the citizen has in their current belief, given their recent failure to find corroboration.

Having calculated this credibility value, the citizen updates their belief distribution via the weighting process described in the Model set-up section above. To reiterate, the citizen's ‘raw’ distribution of the past five information items (i.e. the recent history of information received that has passed evaluation) has the frequency of each item multiplied by two weights: one based on recency, the other on perceived credibility. The sole difference between the calculation occurring during belief updating, and that used during the model set-up, is the credibility weight is not fixed, but rather dynamically calculated for each information item using the relative height of the citizens source credibility distribution (using the left-hand side of equation (5.4)), which is then converted into a weight as follows:

5.7 $$SC_{Weight}=SC_{Height}*100*(CredInf*2),$$

where *CredInf* represents the universal influence of credibility set in the simulation settings (here set to 0.3). In this way, a belief deemed highly credible (e.g. 0.8) will result in a weighting of 48, whilst a belief deemed highly incredible (e.g. 0.2) will result in a weighting of 12. These weights are then used in conjunction with the temporal weight associated with the information items to multiply up the frequency of that information item in the final, processed distribution. As mentioned before, this allows for both the accurate weighting of the distribution mean (i.e. *µ_own_*) by these two factors, but also an appropriate weighting for measures of variance (i.e. *σ_own_*). In the case where no information was found, then the information value is assumed to be both 0.01 and 0.999 for the purposes of calculating the new distribution, as this works to expand the distribution—reflecting the reduction in confidence. These two values are multiplied by a credibility weight using the equation above, with the aforementioned 0.01 inserted value for *SC_Height_*.

It should be noted that whenever this belief distribution update takes place, the overlaid source credibility distribution naturally updates as a function of the former: the SC distribution uses the peak/mean of the (Gaussian) belief distribution as its peak, and takes the standard deviation of the belief distribution as its own, creating a 1:1 correspondence.

Having updated their belief distribution, citizens also then update their confidence in their belief being true (*P*(*H|E*)) using the Bayesian source credibility model [66,67]. This model takes as inputs the prior degree of confidence (i.e. *P*(*H*)) going into this update, and the calculated credibility value associated with this update round (generated via left-hand half of equation (5.4)), and returns a posterior degree of confidence (*P*(*H|E*)) having taken into account the credibility of the latest information. The model performs this update using

5.8 $$P(H|E)=\frac{P(H).\;P(E|H)}{P(H).\;P(E|H)+\;P(\neg H).\;P(E|\neg H)},$$

where *P*(*E*|*H*) = *P*(*E*|*H*, *Exp*, *Trust*) * *P*(*Exp*) * *P*(*Trust*) + *P*(*E*|*H*, ¬*Exp*, *Trust*) * *P*(¬*Exp*) * *P*(*Trust*) + *P*(*E*|*H*, ¬*Exp*, ¬*Trust*) * *P*(¬*Exp*) * *P*(¬*Trust*) + *P*(*E*|*H*, *Exp*, ¬*Trust*) * *P*(*Exp*) * *P*(¬*Trust*); mutatis mutandis for P(E|¬H).

In order to calculate the conditional probabilities, *P(E|H)* and *P(E|*¬*H)*, we follow previous work [68] and integrate perceived trust, *P(Trust)*, and perceived expertise, *P(Expertise)* as an amalgam input, *P(Cred)* – the credibility value associated with the update, for both arguments. The conditional probabilities that dictate the strength of the credibility update on the belief (H) are set as shown in table 1.

**Table 1.** Conditional probability table relating the expected effect of trust (*T*) and expertise (*Exp*) on the updating strength of testimony on the hypothesis (*H*)

evidence	*T*, *Exp*	*T*, ¬*Exp*	¬*T*, *Exp*	¬*T*, ¬*Exp*
*H*	0.8	0.65	0.35	0.2
¬*H*	0.2	0.35	0.65	0.8

These have broad correspondence to previously empirically elicited parameters for the model (see [69]), but provide a working symmetry—given the amalgam (*P*(*Cred*)) of expertise and trust. Values toward 0.5 result correspond to neutral/no influence, while those toward 1 indicate a strong positive influence (increasing update strength positively), and towards 0 a strong negative influence (increasing update strength negatively). As can be seen from table 1, the presence or absence of trust changes the *direction* of the update, while the presence or absence of expertise changes the *strength* of the update. Having performed this update for *P*(*H|E*), the citizen has thus completed their belief and confidence updating for the round.

#### 5.2.6. Information sharing

Any citizens that have made a belief update this round may decide to publicly share information. This can either take the form of sharing the information they themselves received, or of sharing their own updated beliefs (i.e. sharing their opinion). Consequently, this decision-making process has two components to it:

First, if a citizen received information from another source (be it broadcaster or peer), the citizen is able to share this information directly. To do so, the citizen uses their calculation of their perceived credibility of this information (left-hand side of equation (5.4))—which incorporates any independent credibility attached to the source, if that source is a broadcaster—and feeds it into a probabilistic decision-rule, such that the higher the perceived credibility, the more likely the citizen is to share the information directly. If the perceived credibility is higher than a randomly sampled value (uniform between 0 and 0.4), then the citizen has decided to publicly share the information they themselves received, and importantly, the misinformation cues associated with that information. If the information was originally shared by a broadcaster, then any associated independent credibility bonus attached to that broadcaster is also shared, representing any potential source cue effect.

Second, if the citizen is not directly sharing information they received (either because they received none that passed their evaluation, or because they did not deem it sufficiently credible to want to share it publicly), then the citizen uses their current level of confidence that their current belief is correct (*P*(*H|E*)) to decide to share their own opinion, using the same probabilistic decision rule (the higher their confidence the more likely they are to share their opinion). Importantly, in sharing their own opinion (i.e. sharing their *µ_own_* value), the citizen also shares a generated misinformation cue value associated with their post. To generate this value, the citizen samples from a truncated (0–1) normal distribution with a mean corresponding to

5.9 $$abs(Com_{Own}-Truth_{Own})*\alpha_{Citizen},$$

where *Com_Own_* corresponds to the belief of the citizen, *Truth_Own_* corresponds to the average information value of those directly linked network peers of the citizen (who are currently publicly sharing), and *α*_citizen_ corresponds to the proclivity of citizens to engage in misinformation cue deployment. For the present simulations this is set to 0.2, reflecting the lower levels of engagement with misinformation cue deployment [13,14]. In this way, citizens who perceive a more substantial deviation between the belief they are communicating and those they perceive among their peers will be more inclined to use misinformation cues, but this is at a substantially lower level (20%) than the tendency among broadcasters. The standard deviation of this truncated normal distribution for generating the misinformation cue is set to 0.2 in the present simulations (as described in the broadcasting section above), reflecting the general correlation between this perceived deviation purpose behind misinformation cue generation, and the misinformation cue generation itself.

Consequently, having shared this information and attached cues, for information is publicly available to the peers of that citizen, if the citizen decides to share nothing, then other citizens will remain oblivious to their beliefs. This allows for the possibility of deviations between public and private consensus.

#### 5.2.7. Pruning

Lastly, all citizens still active in the network (i.e. those still not completely decided that they are correct; *P*(*H|E*) less than 0.999) prune their local (directly connected) networks. They do this by looking at any of their directly connected peers who are actively sharing information, determining any whose publicly declared information lies outside of the acceptable credibility range of the pruning citizen as no longer credible. This decision-rule uses the credibility cut-off evaluation (equation (5.4)) explained above. Any of the directly connected peers to the pruning citizen deemed no longer acceptable (i.e. no longer credible) are severed from the network of the pruning citizen.

## Data Availability

The model, simulation code, generated datasets and electronic supplementary materials of additional analyses are available at https://osf.io/vjk64/.

## Footnotes

1 We describe the technical and formal components of the model in the Method/technical appendix section. Here, we describe the components informally.

2 Although we only show the medium (0.2) baseline misinformation cue sensitivity condition here, we note that the same pattern is observed irrespective of whether this baseline sensitivity is high or low. The exception to this is a *general* moderating impact of baseline sensitivity on the pattern as a whole, with higher levels of baseline sensitivity producing higher levels of both correct and false-positive rejections.
